# Publication and non-publication of clinical trials in PTSD: an overview

**DOI:** 10.1186/s41073-019-0074-6

**Published:** 2019-07-25

**Authors:** Sharain Suliman, Leigh van den Heuvel, Alexandra Suryapranata, Jonathan I. Bisson, Soraya Seedat

**Affiliations:** 1 Department of Psychiatry, Faculty of Medicine and Health Sciences, Stellenbosch University, PO Box 19063, Cape Town, Tygerberg 7505 South Africa; 2 Division of Psychological Medicine and Clinical Neurosciences, Cardiff University School of Medicine, Cardiff, UK

**Keywords:** Trial registry, Posttraumatic stress disorder, Publication

## Abstract

**Background:**

Although a large number of clinical trials on interventions demonstrating efficacy (or lack thereof) are conducted annually, much of this evidence is not accessible to scientists and clinicians.

**Objectives:**

We aimed to determine the publication rate of posttraumatic stress disorder (PTSD) trials that have been registered in clinical trial registries, and the factors associated with publication.

**Methods:**

Trials, completed on January 15, 2015, were identified via the US National Institutes of Health clinical trials registry, the European Union Clinical Trials Register and the WHO International Clinical Trials Registry Platform. A systematic search for publications (published by the end of March 2018) related to each of the registered trials were then performed.

**Results:**

Four hundred and thirty-eight of 1982 potentially eligible trials were included. Only 34% of interventional trials were registered prior to initiation, 9% were registered within 2 months of starting and 20% after trial completion. Of the 438 included trials, 72% had generated peer-reviewed publications, while an additional 7% had disseminated results in some other form (such as on the trial database), 26 months after trial completion. Randomisation of a trial was the only factor individually associated with publication, in logistic regression analysis (*p* < 0.001). Intervention type, university as sponsor and study registration prior to completion were factors that influenced the time to publication, using Cox regression (*p* < 0.001).

**Conclusions:**

This study underscores the importance of timely and accurate publication and dissemination of trial results, in order to avoid the potential waste of resources and to ensure research integrity and patient safety. We suggest that authors and journal editors adhere to conditions set out by the International Committee of Medical Journal Editors and that more diligent data sharing is encouraged through prospective trial registration and trial reporting websites.

## Background

Although over US$100 billion is invested in medical research, each year reports suggest that less than 50% of funded research is ever published in full [1–5]. There are a number of ethical arguments proposed for why the results of all studies, particularly those involving human volunteers, should be published or reported [6–9]. Turner and colleagues (2013) note that these include the need to increase the returns on public investment (i.e. by the sharing of knowledge and practical advances), increase transparency, prevent research duplication (and wastage of time and resources), allow the public (including participants who have given of their time to the study) access to the research process and the results and inform patient and public decision-making (i.e. through sharing new knowledge and practical advances) [9].

Publication in a peer-reviewed journal is an important form of research dissemination. It offers a record of a study’s scientific contribution, provides evidence to clinicians to inform practice and supports policy makers in decision-making. The scientific community, however, has a poor track record of dissemination of clinical trial results [7, 10]. Poor publication rates may be attributed to a plethora of factors, with some authors struggling to publish data that may not meet the standards of journal editors and readers [7]. Publication bias, leading to lower publication rates for trials with negative or inconclusive results, and delayed publication times are other reasons why research is not published [11–13].

In order to avoid the potential damage that can result from incomplete knowledge and ensure the integrity of research, many organisations have established trial registries as another means to share data. ClinicalTrials.gov was launched on February 29, 2000, initially, with the main aim of establishing a registry of clinical trials investigating drugs for patients with serious or life-threatening diseases, regulated by the Food and Drug Administration (FDA). In 2004, the International Committee of Medical Journal Editors (ICMJE) released a statement that, as of July 2005, journals would require registration with a public trial registry, prior to enrolment, as a condition of publication [14]. In 2006, the World Health Organization (WHO) established standards and rules for the registration of studies in registries, and in 2007, launched the International Clinical Trials Registry Platform (ICTRP), which brings together data from all registries meeting their standards into a single searchable portal [15]. Researchers are currently required to register interventional trials while the registration of observational studies is optional [16]. The Declaration of Helsinki, since 2008, re-stipulates that research studies involving human subjects must be registered in a publicly accessible database before recruitment of the first subject and further states that researchers, authors, sponsors, editors and publishers all have ethical obligations with regard to the publication and dissemination of the results of their research [17]. Clinicaltrials.gov and the European Clinical Trials Database (EudraCT) have each included a section that permits investigators to submit their results, since 2008 and 2013, respectively.

Recent advances in reporting requirements include the Department of Health and Human Services (HHS) final rule, which clarifies and expands the requirements for reporting results information [18]. This was made publicly available on September 16, 2016. The NIH, concurrently, issued a complementary policy under which all NIH-funded clinical trials are expected to submit registration and results’ information, whether or not the trials are covered by the FDA Amendments Act requirements. This NIH Final Rule (42 CFR Part 11) came into effect on January 18, 2017, with compliance expected on April 18, 2017 [19]. Further, the American Psychological Association’s (APA’s) revised reporting standards for writing journal articles were released. This calls for registration of a study prior to its implementation and a summary of results on completion [20].

We examined the publication of all registered and completed posttraumatic stress disorder (PTSD)-related intervention trials in the ICTRP and in two major trial registries (ClinicalTrials.gov and EudraCT), in order to determine the rate of and time to publication and factors associated with publication. In order to be as inclusive as possible, we included trials where individuals with PTSD were included or where any PTSD-related outcomes (e.g. diagnosis, severity, symptomatology) were assessed. PTSD is a highly comorbid disorder and many of the drug and psychological interventions investigated in trials have trans-diagnostic application and utility.

In this paper, we address the following questions:

(i) What proportion of PTSD-related intervention trials registered in ClinicalTrials.gov, EudraCT and ICTRP go on to be published and what are the characteristics of these studies?
(ii) What is the time to publication and what factors pertinent to registration are associated with time to publication?

We hypothesised the following:

(i) Proportion of and time to publication of PTSD-related studies would be similar to other conditions/disciplines and that individual factors related to study quality and characteristics would influence publication rates and times (we did not have pre-specified hypotheses as to what these individual factors would be).
(ii) Publication rates of studies with positive (statistically significant) outcomes would be higher than those with null (not statistically significant) outcomes.

Outcomes were as follows:

(i) Proportion of completed intervention trials that go on to be published.
(ii) Length of time between study completion and publication.
(iii) Factors that contributed to publication.

## Methods

**Fig. 1:**
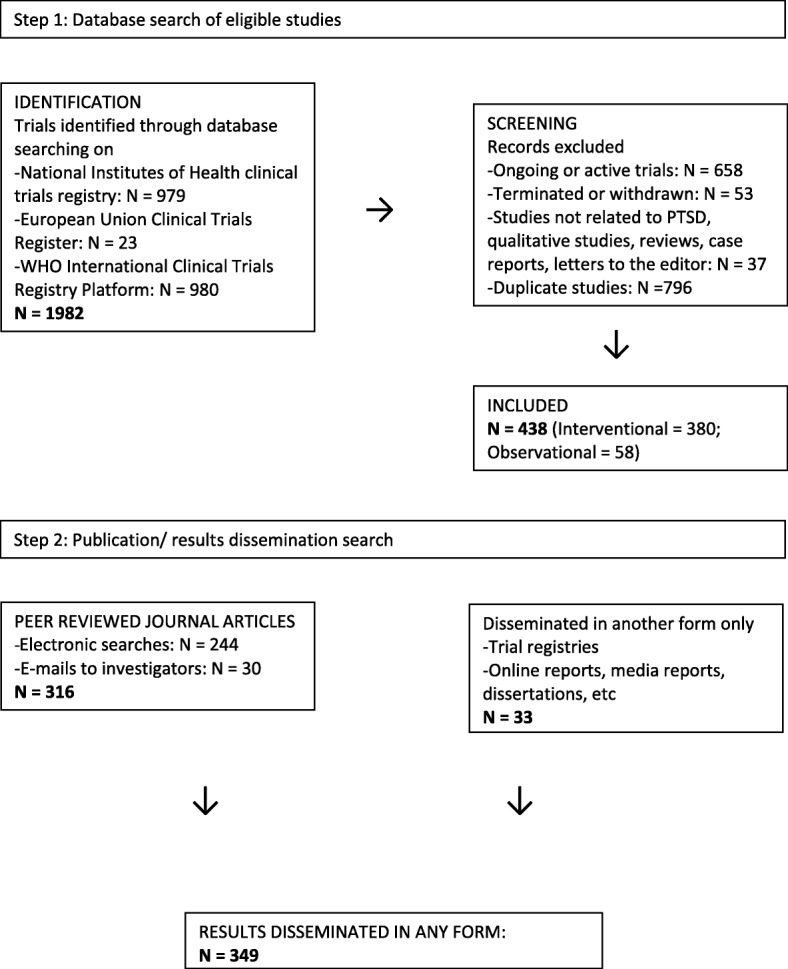
Flow diagram of the selection process

This was a cross-sectional overview of registered and subsequently published trials. Considering that this was an overview that entailed pooled analysis of secondary data, ethical approval was not sought. Two independent researchers (SS1 and LvdH) selected studies through each phase of selection. The responsible researchers agreed on the search terms to use and on study selection and exclusion criteria, as well as on data abstraction (selection of data items extracted from each contributing article) and data extraction. The search terms included all applicable synonyms for PTSD, were compiled in accordance with the instructions for each database and were tested prior to implementation. The exact search terms used were “PTSD OR posttraumatic stress disorder OR post-traumatic stress disorder OR posttraumatic stress disorder.” In each of the registries and search platform, we searched all fields and placed no limits on the searches. Our search terms were broad as we aimed to identify all possible PTSD-related studies. Studies were identified via Clinicaltrials.gov (www.clinicaltrials.gov), EudraCT (https://www.clinicaltrialsregister.eu/) and ICTRP (http://apps.who.int/trialsearch/Default.aspx). We extracted data on all studies and manually identified studies that were closed or completed. The ICTRP is a search platform containing trial registration data obtained from primary registries and partner registries internationally that meet the WHO clinical registry standards. We selected these three registries/platforms because they are the largest. Trials registered since the inception of each registry were downloaded on January 15, 2015; therefore, the date range for the NIH registry was on February 29, 2000, until January 15, 2015, for the EU register on May 1, 2004, until January 15, 2015, and for the WHO platform on May 1, 2007, until January 15, 2015. Inclusion criteria were predefined. Any PTSD-related study, with original quantitative data, was included. Qualitative studies, reviews, case reports and letters to the editor were excluded (although we did not expect to find these other study types registered on the trial registries as they do not clearly fall within the purpose of trial registries, we did). After ongoing or terminated trials, duplicates and studies not related to PTSD were removed, and there were 438 records to review. (Terminated trials were excluded as they are less likely to publish results, and our main aim was to evaluate the publication of completed studies.) See Fig. 1. We obtained independent peer review and approval of the search strategy and documentation by an information specialist who utilises the PRESS methodology.

Information of interest pertaining to each trial was systematically extracted from the databases by two researchers (SS1 and LvdH) and reconciled. This included the following:

1. Database registration (which database/platform, when registered)
2. Study status (completed, terminated/withdrawn, active)
3. For completed studies: type of study (interventional, observational, study design); intervention type (i.e. behavioural/ psychological, such as cognitive behavioural therapy, narrative exposure therapy, motivational interviewing, mindfulness-based therapy; drug, device/procedure, such as transcranial direct current stimulation, photobiomodulation, biofeedback), study duration, number of arms, control conditions, location, numbers planned vs enrolled; participants (types, numbers in each arm); time between end of trial and publication; study sponsors as defined in the ICTRP^1^ (none, pharmaceutical/industry, government, non-commercial, hospital, university); date and phase of study registered; study outcome^2^ (primary and secondary outcomes); changes to trial information, contact details of responsible party
4. Publication: in a journal or disseminated in another form

Five investigators then searched for peer-reviewed publications or any results disseminated for each individual study that was registered (LvdH and SS1 conducted the initial search and data extraction, which were verified by AS; IE and SW updated the results which were checked by SS1). For each registered trial, we searched via PubMed, Scopus, Google Scholar and Google using keywords listed (when available), trial titles, investigator names and trial registration codes to identify matching publications and/or results. Any report of a trial that had included results for PTSD as a primary or secondary outcome or as a covariate or had sampled individuals with PTSD was included. We considered a trial *published* if it appeared in a peer-reviewed journal (whether or not we could obtain the full article). We considered trial results *disseminated* if they appeared in a peer-reviewed journal, in a trial registry, in a report (e.g. media article, organisation website) or thesis. If publications were not found, investigators of these completed trials were e-mailed to ask for study-related publications and results (71 e-mail addresses were obtained via the registries and 96 via online searches; we were unable to obtain e-mail addresses for 27 investigators. Of the 167 investigators who were e-mailed, 70 responded). If the completion date of a trial was missing (5% of trials), we used the publication date as a proxy when this was available. This search was completed on March 21, 2018, which yielded a minimum of 26 months between trial completion (according to the completion date listed in the trial registries) and publication. We describe all studies in the results, but given the small number of observational studies listed and the fact that registration for these studies is voluntary (they are not required to be registered), we excluded them from further analysis.

Publications and trial data were independently reviewed by two investigators to determine whether (i) manuscripts or reported results were in fact related to the trial listed in the registry (as not all manuscripts/reports noted the trial registration number in the publication, it was not always clear, so a second reviewer double-checked this), (ii) PTSD-related findings were reported (e.g. some reports/papers reported on other study findings, but omitted to report findings related to PTSD—PTSD might have been a secondary outcome in some of these), (iii) PTSD-related outcomes were the primary or secondary outcomes measured, both or other (e.g. a condition or covariate) and (iv) whether primary and secondary findings were positive (statistically significant, *p* < 0.05), null (no statistically significant finding, *p* ≥ 0.05) or mixed (both statistically significant and no statistically significant findings in primary and/or secondary outcomes). Where results were reported in both a publication and another disseminated source and these differed, the publication results were used.

## Statistical analysis

We present descriptive statistics for all studies split according to interventional and observational trials. As our main outcomes of interest were related to interventional trials, we examined univariate factors associated with the publication of interventional trials and entered significant variables into a logistic regression. The Kaplan-Meier method [21] was used to estimate the time between intervention trial completion and a paper related to that trial being published in a journal while the Breslow statistic [22] was used to determine significance. Significant covariates were then entered into a Cox Regression [23] to determine whether the time to publication was predicted by significant covariates. Analyses were conducted in SPSS, with two-tailed tests. *p* values of ≤ 0.05 were considered significant.

## Results

### Descriptive results of trials from the registry

All 438 trials were found on the ICTRP search platform, although 53 (12%) were not initially identified via ICTRP with the search terms used. Of these, 52 (12%) were identified in ClinicaTrials.gov only and 1 (0.2%) on EudraCT only. Furthermore, 322 (74%) trials were identified in both ICTRP and ClinicalTrials.gov, 2 (0.5%) in ICTRP and EudraCT and 61 (14%) in ICTRP only. In terms of registries of primary origin, 381 (87%) were registered in ClinicalTrial.gov; 21 (5%) on BioMed Central’s International Standard Registered Clinical/soCial sTudy Number (ISRCTN); 9 (2%) in the Australian New Zealand Clinical Trials Registry (ANZCTR); 8 (2%) in the Iranian Registry of Clinical Trials (IRCT), with 5 or less from Japan Primary Registries Network (JPRN) (1%); Chinese Clinical Trials Registry (ChiCTR) (< 1%); EudraCT (< 1%); German Clinical Trials register (< 1%); Netherlands Trial register (< 1%); and Clinical Trials Registry - India (CTRI) (< 1%).

The majority of included trials, 380 (87%) were interventional and 58 (13%) were observational in design. [See Table 1]. According to the data downloaded from the trial registries, PTSD was the *primary condition* in 91% of studies, *main outcomes* were PTSD related in 69% of trials, and *secondary outcomes* were PTSD related in 34% of trials. Around two thirds (66%) of studies were conducted in North America, followed by Europe (15%), Asia (13%), Africa (2%), Australia (2%) and South America (1%). Only 4.2% of studies were registered in low- and middle-income countries (based on the Word Bank categories).

**Table 1:** Descriptive data for interventional and observational trials listed in registries

Variable		Total	Interventional	Observational
		*n* = 438	*n* = 380	*n* = 58
Number enrolled, Mdn (IQR)		70 (36; 133)	62 (33; 120)	209 (78; 500)
Duration of study (months), Mdn (IQR)		29 (16; 45)	31 (16; 47)	26 (13; 36)
Gender, *n* (%)				
	Male only	35 (8)	30 (8)	5 (9)
	Female only	54 (12)	49 (13)	5 (9)
	Both	348 (80)	300 (79)	48 (83)
Age group				
	Adult	383 (87)	331 (87)	52 (89)
	Child	37 (8)	33 (9)	4 (6)
	Both	18 (4)	16 (4)	2 (3)
Any control condition used, *n* (%) (e.g. waitlist, placebo)		354 (81)	339 (89)	15 (26)
Comparator used		136 (31)	127 (33)	9 (16)
Randomisation, *n* (%)				n/a
	Randomised		326 (86)	
	Non-randomised		21 (6)	
	Single group		28 (7)	
	Unclear		5 (1)	
Blinding				n/a
	Double blind		103 (27)	
	Single blind		108 (28)	
	Open label		132 (35)	
	Unclear		37 (10)	
Intervention type, *n* (%)				n/a
	Drug		85 (22)	
	Behavioural		247 (65)	
	Drug and behavioural		25 (7)	
	Procedure/device		23 (6)	
Number of study arms, *n* (%)				
	One	73 (17)	40 (11)	33 (57)
	Two	294 (67)	278 (73)	16 (28)
	Three to six	71 (16)	62 (16)	9 (16)
Actual vs planned enrolment, *n* (%)				
	More than planned	71 (16)	57 (15)	14 (24)
	Less than planned	171 (43)	150 (37)	21 (36)
	No difference	187 (39)	168 (44)	19 (33)
Status of main sponsor, *n* (%)				
	University	176 (40)	164 (43)	12 (21)
	Hospital	93 (21)	76 (20)	17 (29)
	Governmental organisation	114 (26)	90 (24)	24 (41)
	Non-commercial (e.g. NGO)	47 (11)	44 (12)	3 (5)
	Pharmaceutical/industry	8 (2)	6 (2)	2 (3)
Any secondary sponsors listed, *n* (%)		209 (48)	193 (51)	16 (28)
Any sponsor university		199 (45)	183 (48)	16 (28)
Any sponsor hospital		106 (24)	89 (23)	17 (30)
Any sponsor governmental organisation		231 (53)	200 (53)	31 (54)
Any sponsor non-commercial		72 (17)	67 (18)	5 (9)
Any sponsor pharmaceutical/industry		34 (8)	29 (8)	5 (9)
Contact email provided, *n* (%)		188 (43)	172 (45)	16 (28)
Registered before 2006, *n* (%)		97 (22)	81 (21)	16 (28)
Study registered before started, *n* (%)		151 (35)	127 (34)	24 (42)
Study registered before completed, *n* (%)		336 (80)	291 (80)	45 (79)

*More/less than planned was based on a difference of 10% or more and obtained from the trial registry. Any sponsor/secondary sponsors include university, hospital, governmental or pharmaceutical/industry funding.*

*IQR* interquartile range, *Mdn* median*, NGO* non-governmental irganization, *n* number; *%* percentage

*p < 0.05

Blinding status varied significantly (*p* < 0.001) according to intervention type with drug trials more likely to be double-blind (73%) than open-label (19%). Psychological intervention trials were more likely to be open-label (43%) followed by single-blind (37%), and combined drug and psychological intervention trials were more likely to be double-blind (60%) than single-blind (28%).

Pharmaceutical companies and other industry accounted for the smallest sponsorship contribution (8%). Industry/pharmaceutical companies were more likely (*p* < 0.001) to be a sponsor of drug trials (79%), followed by sponsorship of trials of psychological interventions (17%) and combined psychological and drug interventions (3%). Universities were more likely (*p* = 0.001) to be sponsors of psychological intervention trials (74%), followed by drug (19%) and combined psychological and drug trials (6%). Hospitals were more likely (*p* = 0.024) to be a sponsor of psychological intervention trials (56%), followed by drug trials (24%), trials of procedures/devices (12%) and combined psychological and drug intervention trials (8%). Governmental organisations were also more likely (*p* = 0.034) to sponsor psychological intervention trials (71%), followed by drug (17%), combined drug and psychological intervention trials (7%) and studies of procedures/devices (5%). There was no significant relationship between non-commercial sponsorship and the type of intervention.

Entries into the database were changed over time, with evidence that information entered was updated or changed at least once in 82% of the studies. Registration rates increased after registration was made a requirement by the ICJME—37% of trials were registered prior to initiation in 2006 or later versus 20% in the preceding years (*p* = 0.04).

### Published and disseminated result outcomes

Of the trials that were registered, 317 (74%) yielded traceable peer-reviewed publications, while another 32 (8%) studies had disseminated results in some other form (e.g. on the trial registry, in a dissertation or published report) (Table 2). Findings were obtained for 308 (81%) interventional trials. We were unable to obtain the remaining papers that had been disseminated, and thus, unable to extract the results.

**Table 2:** Publication data for (i) all studies and (ii) interventional trials

Variable	Total	Interventional
Results disseminated in any form, *n* (%)	349 (80)	313 (82)
Results found on registry, *n* (%)	103 (24)	96 (25)
Published in a journal, *n* (%)	317 (72)	281 (74)
Obtained results for the trial, *n* (%)	343 (78)	308 (81)
Are the results related to PTSD, *n* (%)	326 (95)	298 (97)
PTSD diagnosis/symptoms as an outcome, *n* (%)		
Primary	170 (52)	160 (54)
Secondary	57 (18)	51 (17)
Both primary and secondary	58 (18)	55 (19)
Other (e.g. covariate, condition studied)	40 (12)	31 (10)
Primary outcome measure/s, *n* (%)		
Positive	181 (59)	165 (59)
Null	75 (25)	67 (24)
Mixed	50 (16)	47 (17)
Secondary outcome measure/s, *n* (%)		
Positive	144 (44)	111 (43)
Null	57 (20)	55 (21)
Mixed	98 (35)	92 (36)

### Factors associated with publication of interventional trials

Whether a trial was randomised (*p* = 0.002), the type of intervention studied (*p* = 0.010), whether a pharmaceutical company/industry was involved in sponsoring the trial (*p* = 0.005) and whether a contact email address was provided in the registry (*p* = 0.021), were factors that were significantly associated with a trial being published in a journal (Table 3). These factors were subsequently entered into a logistic regression.

**Table 3:** Factors associated with publication of interventional trials

Variable	Published	Not published	Test statistic	*p* value
Randomised, *n* (%)	252 (90)	74 (78)	*χ*^2^(1) = 9.15	< 0.002*
Blinding			*χ*^2^(2) = 5.51	0.064
Double blind	78 (31)	25 (28)		
Single blind	86 (34)	22 (24)		
Open label	88 (35)	44 (48)		
Intervention type, *n* (%)			*χ*^2^(3) = 11.45	0.010*
Drug	52 (19)	33 (33)		
Behavioural	191 (68)	56 (57)		
Drug and behavioural	22 (8)	3 (3)		
Procedure/device (e.g. TMS)	16 (6)	7 (7)		
Any control condition used, *n* (%) (e.g. waitlist, TAU)	255 (91)	84 (85)	*χ*^2^(1) = 2.65	0.104
Placebo used	68 (24)	27 (27)	*χ*^2^(1) = 0.369	0.544
Comparator used	94 (34)	33 (33)	*χ*^2^(1) = 0.00	0.983
Number of study arms, *n* (%)			*χ*^2^(2) = 5.35	0.069
One	24 (9)	16 (16)		
Two	213 (76)	65 (66)		
Three to six	44 (16)	18 (18)		
Status of the main sponsor, *n* (%)			*χ*^2^(4) = 9.3	0.055
University	128 (46)	36 (36)		
Hospital	59 (21)	17 (17)		
Governmental organisation	57 (20)	33 (33)		
Non-commercial (e.g. NGO)	34 (12)	10 (10)		
Pharmaceutical/industry	3 (1)	3 (3)		
Any secondary sponsor listed, *n* (%)	148 (53)	45 (46)	*χ*^2^(1) = 1.53	0.217
Any sponsor university	141 (50)	42 (42)	*χ*^2^(1) = 1.76	0.184
Any sponsor hospital	71 (25)	182 (18)	*χ*^2^(1) = 2.049	0.152
Any sponsor governmental organisation	146 (52)	54 (55)	*χ*^2^(1) = 0.20	0.657
Any sponsor non-commercial	53 (19)	14 (14)	*χ*^2^(1) = 1.12	0.289
Any sponsor pharmaceutical/industry	15 (5)	14 (14)	*χ*^2^(1) = 8.048	0.005*
Contact email provided, *n* (%)	137 (49)	35 (35)	*χ*^2^(1) = 5.31	0.021*
Are the results related to PTSD, *n* (%)	269 (97)	29 (97)	*χ*^2^(1) = 0.00	0.978
PTSD-related outcome/s, *n* (%)			*χ*^2^(3) = 4.75	0.191
Primary	145 (54)	15 (54)		
Secondary	47 (18)	4 (14)		
Both primary and secondary	52 (19)	3 (11)		
Other (e.g. covariate or condition studied)	25 (9)	6 (21)		
Primary outcome measure/s, *n* (%)			*χ*^2^(2) = 5.64	0.060
Positive	156 (59)	9 (56)		
Null	60 (23)	7 (44)		
Mixed	47 (18)	0 (0)		
Secondary outcome measure/s, *n* (%)			*χ*^2^(2) = 1. 65	0.439
Positive	109 (44)	2 (25)		
Null	52 (21)	3 (38)		
Mixed	89 (36)	3 (38)		
Study registered before started, *n* (%)	92 (33)	35 (35)	*χ*^2^(1) = 1.17	0.683
Study registered before completed, *n* (%)	207 (78)	84 (86)	*χ*^2^(1) = 2.42	0.120

*p < 0.05

The overall model was significant (*χ*^2^ (6) = 22.26, *p* < 0.001), with a Nagelkerke *R*^2^ of 0.085, and predicted 75% of publications correctly. The only variable that was individually associated with publication status was randomisation (*p* = 0.007). Having pharmaceutical/industry sponsorship (*p* = 0.073), provision of a contact email (*p* = 0.088) and intervention type were not significant (Table 4).

**Table 4:** Logistic regression of factors influencing publication

Variable	*B*	Wald *χ*^2^	Significance	Odds ratio (95% CI)
Constant	− 0.11	0.07	0.790	
Randomised	0.87	7.20	0.007*	2.40 (1.27; 4.54)
Intervention type				
Drug^a^		3.21	0.360	
Behavioural	0.41	1.73	0.189	1.50 (0.82; 2.76)
Procedure/device	0.31	0.30	0.584	1.36 (0.45; 4.12)
Drug and behavioural	1.07	2.52	0.112	2.92 (0.78; 10.93)
Any sponsor pharmaceutical/industry	0.79	3.21	0.073	0.45 (0.19; 1.08)
Contact email provided	0.43	2.92	0.088	1.54 (0.94; 2.53)

Model *χ*^2^(6) = 22.26 (*p* < 0.001*)

Nagelkerke *R*^2^ = 0.085

*Significance set at *p* < 0.05

^a^Drug intervention was the type of intervention against which other interventions were analysed

### Factors associated with time to publication of interventional trials

The Kaplan-Meier method [21] was used to estimate the time between trial completion and a trial-related paper being published (Table 5). The median time to journal publication was 27.0 months (95% CI 24.0; 30.0). Factors that influenced time to publication of interventional trials were whether a control condition was used (*p* = 0.021), whether the trial was randomised (*p* = 0.013), the intervention type (*p* = 0.004), whether a university was involved as a sponsor (*p* = 0.026), the number of treatment arms (*p* = 0.034) and whether the study was registered in a trial registry before it was completed (*p* = 0.040).

**Table 5:** Time to publication for interventional trials

Variable	Median survival time (95% CI)	Chi-square	*p* value
Overall	27 (24; 30)		
Randomised		*χ*^2^(1) = 6.15	0.013*
Yes	26 (23; 29)		
No	39 (32; 46)		
Blinding		*χ*^2^(2) = 2.54	0.281
Double blind	30 (24; 36)		
Single blind	24 (21; 27)		
Open label	23 (26; 40)		
Intervention type		*χ*^2^(3) = 13.33	0.004*
Drug	39 (28; 50)		
Behavioural	26 (23; 29)		
Procedure/device(e.g. TMS)	27 (24; 31)		
Drug and behavioural	20 (20; 34)		
Any control condition used (e.g. waitlist, TAU)		*χ*^2^(1) = 5.35	0.021*
Yes	26 (23; 29)		
No	39 (33; 45)		
Placebo used		*χ*^2^(1) = 0.65	0.422
Yes	30 (24; 37)		
No	27 (24; 30)		
Comparator used		*χ*^2^(1) = 0.97	0.324
Yes	27 (23; 31)		
No	29 (24; 34)		
Number of study arms		*χ*^2^(2) = 6.75	0.034*
One	44 (37; 52)		
Two	25 (22; 28)		
Three to six	30 (20; 40)		
Any sponsor university		*χ*^2^(1) = 4.99	0.026*
Yes	24 (20; 28)		
No	32 (26; 38)		
Any sponsor hospital		*χ*^2^(1) = 0.08	0.776
Yes	29 (21; 37)		
No	27 (24; 30)		
Any sponsor governmental organisation		*χ*^2^(1) = 0.450	0.502
Yes	28 (24; 32)		
No	26 (22; 30)		
Any sponsor pharmaceutical/industry		*χ*^2^(1) = 2.896	0.086
Yes	40 (0; 83)		
No	27 (24; 31)		
Any sponsor non-commercial		*χ*^2^(1) = 0.90	0.344
Yes	34 (23; 45)		
No	27 (24; 30)		
Contact email provided		*χ*^2^(1) = 0.87	0.352
Yes	26 (23; 29)		
No	29 (24; 34)		
Are the results related to PTSD		*χ*^2^(1) = 0.26	0.613
Yes	27 (23; 31)		
No	39 (1; 77)		
PTSD-related outcome		*χ*^2^(3) = 1.80	0.615
Primary	31 (22; 40)		
Secondary	26 (23; 29)		
Both primary and secondary	25 (19; 31)		
Other (e.g. covariate, condition studied)	30 (22; 38)		
Primary outcome measure/s		*χ*^2^(2) = 2.32	0.115
Positive	26 (23; 29)		
Null	31 (25; 37)		
Mixed	23 (14; 32)		
Secondary outcome measure/s		*χ*^2^(2) = 1.50	0.473
Positive	25 (21; 29)		
Null	29 (22; 36)		
Mixed	24 (20; 28)		
Registered before 2006		*χ*^2^(1) = 0.32	0.573
Yes	31 (24; 39)		
No	27 (24; 30)		
Study registered before started		*χ*^2^(1) = 1.07	0.302
Yes	27 (23; 31)		
No	28 (24; 32)		
Study registered before completed		*χ*^2^(1) = 4.22	0.040*
Yes	28 (24; 32)		
No	35 (26; 44)		

*Significance set at *p* < 0.05

These significant factors were entered into a Cox regression (Table 6). The overall model was significant (*χ*^2^ (9) = 32.69, *p* < 0.001) with a 2 log-likelihood of 2271. Variables influencing publication time were intervention type—drug-only studies took significantly longer to publish than any other intervention study type; university sponsorship was associated with faster publication time; and trials that were registered in a database before being completed were also associated with faster publication times. Randomisation, number of treatment arms and having a control group were no longer statistically significant.

**Table 6:** Cox regression of factors involved in publication time

Variable	*B*	Wald *χ*^2^	Significance	Relative risk (95% CI)
Randomised	− 0.21	0.20	0. 653	0.82 (0.33; 1.99)
Treatment arms^a^		1.87	0.392	
2 arms	0.55	0.87	0.351	1.74 (0.54; 5.56)
3–6 arms	0.36	0.34	0.562	1.43 (0.43; 4.73)
Any control used	0.095	0.07	0.797	1.10 (0.53; 2.27)
Intervention type^b^		18.64	< 0.001*	
Behavioural	0.51	8.65	0.003*	1.67 (1.19; 2.34)
Drug and behavioural	1.16	16.79	< 0.001*	3.20 (1.84; 5. 58)
Procedure/device	0.74	5.54	0.019*	2.09 (1.13; 3.87)
Any sponsor, university	0.26	3.88	0.049*	1.30 (1.00; 1.70)
Registered before completed	0.33	4.10	0.043*	1.40 (1.01; 1.93)

Model χ2(9) = 32.69 (*p* < 0.001*)

− 2 log-likelihood = 2271

*Significance set at *p* < 0.05

^a^One arm was the number of arms against which other arms were analysed

^b^Drug intervention was the type of intervention against which other interventions were analysed

## Discussion

We conducted a review of trials related to PTSD in the ICTRP platform and two of the largest trial registry databases with a view to determining factors that might influence publication. Of the 380 registered interventional trials included, we found that at least 26 months after trial completion, the peer-reviewed journal publication rate was 74%. This increased to 82% when we included trials that had published results in some other form (e.g. in a trial registry or in a published report). This is better than or equivalent to the rate reported for other disease areas and is likely to further increase with time since trial completion. In one study on vaccine trials, the publication rate after 12 months was 12% and increased to 73% at 48 months post-study completion [24]. Another study of randomised clinical trials registered with ClinicalTrials.gov reported a publication rate of 71% at 60 months after study completion [25]. Time from the close of a study until publication was approximately 2 years and 3 months. This is in line with findings from other studies [26, 27], but may be longer given that when the completion date of a trial was missing, we used the publication date as a proxy.

It is concerning that even though registration prior to enrolment is required by the ICMJE, and has been included in the Declaration of Helsinki, only one third of interventional trials were registered prior to initiation, a further 9% within 2 months of starting and 20% after trial completion. Furthermore, despite the rate of registration prior to trial initiation having increased over time, the rate since 2006 is much lower than expected (over 60% were registered after trial initiation), when considering that the ICMJE instituted its requirement in 2005. A recent review of articles published in psychiatry journals which require prospective trial registration found that 34% were retrospectively registered and that only 33% of studies were correctly prospectively registered [28]. Another study found that only 54% of recently published antidepressant and cognitive behavioural therapy trials were registered and only 25% were properly registered [29]. Only 15% of RCTs published in clinical psychology journals were registered prospectively and even fewer (1%) were both prospectively and completely registered [30]. Similarly, the large number of trials with no documented difference in the number of participants planned for enrolment and the number actually enrolled is likely due to studies that were registered retrospectively. As clinical trial registries are used by researchers, clinicians and the public, it is important that they are accurate and up to date. Our findings also highlight the dearth of trials registered in low- and middle-income countries. Studies from these countries may be registered in trial databases not included in this review, but as the ICTRP only includes registries fulfilling their standards, these databases may not meet current requirements.

Trial randomisation was the only factor associated with peer-reviewed publication, when other factors were accounted for. Although not all prior studies have found that more rigorous trial designs (i.e. randomisation) are associated with publication [13], our results indicate that they could be. Although only six interventional trials had primary pharmaceutical/industry sponsorship and 29 had some industry involvement, these trials trended towards a lower likelihood of publication. Given that the industry provided sponsorship for 79% of drug-only trials, this finding suggests that sponsorship may influence publication with journals/industry setting stricter criteria for drug trials. Provision of a contact e-mail and intervention type was not significant when these other variables were accounted for. Whether results were positive or null was also not significantly associated with publication status. This finding, however, does need to be interpreted with caution as it is likely influenced by the number of unpublished trials for which we were unable to obtain any data.

With regard to the time to publication of interventional trials, trials of drug interventions alone were likely to be published later than those of drug and psychological interventions, psychological interventions alone and those assessing a procedure or device. Again, this may be because journals and/or authors have stricter publication requirements for drug trials. Nonetheless, this is concerning given the potentially serious and far-reaching implications of a lack of up-to-date evidence on the provision of effective pharmacotherapies to inform treatment guidelines. Trials with a university involved as a sponsor were likely to be published sooner than those without. This may be due to universities providing funding to their own academics, who are often rewarded based on their publication outputs. Trials registered in a trial database before trial completion were also likely to be published sooner than those registered after completion. This may indicate that authors are likely to consider and prepare their data for publication prior to trial completion and that those who register their trials before trial closeout intend to publish their data. Trial randomisation was no longer statistically significant when these other factors were accounted for.

Some of the authors we contacted provided reasons for not having published their work. These included results not worth publishing, small sample size, null findings and publication rejected by journals submitted to. Other reports have noted similar motivations for non-publication. Publication bias and lower publication rates for studies with negative or inconclusive results include the high standards of journal editors and the desire for positive results [7, 9, 11]. It has, however, been pointed out that the bias towards positive findings may come from the authors themselves, rather than journal editors and reviewers.

The strengths of this study are that it considered a large number of trials that encompassed a variety of research methodologies and were registered across a number of databases. To our knowledge, this is the first to do this in the field of psychiatry and PTSD. Despite our focus on PTSD-related trials, our results support the reports of publication bias in other fields [24, 29]. It is, however, important to recognise that there have been major developments in trial reporting regulations and guidelines in recent years (42 CFR Part 11, The Final Rule, APA’s revised JARS guidelines). Thus, trials conducted prior to these may be unrepresentative of current registration and reporting practices. Nonetheless, our findings support these new registration and reporting requirements and indicate the need for mechanisms (i.e. policies to support registration and reporting with penalties for investigators who do not follow standards for clinical research [31]) that ensure these regulations are adhered to, as we have demonstrated that since the ICMJE regulations many trials were still only registered after completion.

The following limitations should also be noted: (i) incorrect, inconsistent and incomplete information listed in the trial registries. For example, we found that information was not updated regularly, information in one section of the registry did not always align with others and trials that appeared on the list of “complete” studies were at times still listed as “active.” This may also imply that we may have missed trials that were in fact completed, but were not updated as such on the system. This has been a drawback since the inception of the registries [32]. There may also be some misclassification of hospital and university sponsors. We based this on the name of the sponsor as entered in the trial registration database. In some instances, university hospitals may be the primary sponsor but the university may, in fact, be listed as the sponsor. We performed a post hoc analysis to assess whether the effect of university sponsorship on time to publication was independent of hospital sponsorship by adding hospital sponsorship to the model and university sponsorship remained significantly associated with time to publication, whereas hospital sponsorship was not; (ii) although we made every effort to locate published reports—peer-reviewed and other, we may not have traced all, as publications were not always linked to trial registration numbers and not all study contacts listed responded; (iii) we included various study types, not only intervention trials in our initial analyses. Given that only intervention trials are required to be listed on these registries, we opted to exclude the observational studies from further analysis. This, however, constituted only a small percentage of studies; (iv) similarly, there were only a small number of trials sponsored by industry, which warrants care in the interpretation of the related findings; (v) we attempted to retrieve publications after a minimum of 26 months post-completion. It is possible that our publication rate would have increased had we used a longer post-completion timeframe; (vi) although the search strategies were the same across databases/search portals, not all the studies were identified in ICTRP. This may be related to differences in search algorithms; (vii) these results may not be generalizable to all trials. For example, previous studies suggest that the publication rate for registered trials is higher than for other trials [33]. Similarly, registered trials might differ from trials initially published as abstracts (e.g. Scherer et al., 2018) or trials identified through Internal Review Boards (e.g. Chan et al., 2004) [5, 34]. Furthermore, registered trials may differ systematically from unregistered trials, confounding interpretation of factors associated with journal publication. Drugs and devices, for instance, are FDA-regulated products and, as such, these trials are much more likely to be registered and also more likely to be reported in biomedical journals (which are often ICMJE members and have registration requirements). Trials of non-regulated products, on the other hand, are more likely to be reported in psychology and allied health journals (which are not ICMJE members and do not have registration requirements). This is illustrated by a study that reviewed the registration of clinical trials in clinical psychology journals, which found that most clinical trials in psychology journals (with the exception of registered trials in clinical psychology) were not registered [30].

In sum, 74% of trials were published in peer-reviewed journals and a further 8% had published results in some other form 26 months after trial completion; publication time from trial completion was approximately 2 years and 3 months; despite ICMJE guidelines, a little over one third of interventional trials were registered prior to initiation; and few trials were registered in low- and middle-income countries. Randomised trials were more likely to be published and those with a contact e-mail and no pharmaceutical sponsorship were not significant when other variables were accounted for; null findings were not associated with publication status. Trials sponsored by a university and those registered in a trial database before trial completion were likely to be published sooner than those registered after completion, while drug-only trials took longer to publish.

## Conclusion

By not sharing knowledge gained from these trials, researchers waste valuable research funds, slow down advancement in the field, put patients and study participants at risk of potentially ineffective or even harmful interventions and distort the evidence base [11, 13]. In order to safeguard research integrity, we therefore support the call to encourage and make mandatory other methods of data sharing, for example via prospective trial registration and reporting websites such as those that we examined. Furthermore, given that the utility of information on registries is dependent on the quality of the information entered by investigators, the large amount of inconsistent or missing information we found on the databases indicates that greater vigilance is required to improve the reliability and accuracy of reporting. Recent initiatives in trial reporting regulations are a step towards this.

## Data Availability

The datasets used and/or analysed during the current study are available from the corresponding author on reasonable request.

## Abbreviations

%: Percentage
ANZCTR: Australian New Zealand Clinical Trials Registry
APA: American Psychiatric Association
ChiCTR: Chinese Clinical Trials Registry
CTRI: Clinical Trials Registry - India
EU: European Union
EudraCT: European Clinical Trials Database
FDA: Food and Drug Administration
HHS: Department of Health and Human Services
ICMJE: International Committee of Medical Journal Editors
ICTRP: International Clinical Trials Registry Platform
IQR: Interquartile Range
IRCT: Iranian Registry of Clinical Trials
ISRCTN: International Standard Registered Clinical/soCial sTudy Number
JPRN: Japan Primary Registries Network
Mdn: Median
*n*: Number
NGO: Non-governmental Organisation
NIH: National Institutes of Health
PTSD: Posttraumatic stress disorder
WHO: World Health Organization

## Footnotes

1 Sponsor refers to the individual, organization, group or other legal entity which takes responsibility for initiating, managing and/or financing a study. The Primary Sponsor may or may not be the main funder.

2 Outcome or outcome measure is defined as measurements observed or collected from those human subjects who are enrolled in a research trial.
